# SMYD2 promotes tumorigenesis and metastasis of lung adenocarcinoma through RPS7

**DOI:** 10.1038/s41419-021-03720-w

**Published:** 2021-05-02

**Authors:** Lei Wu, Fan Kou, Zhenyu Ji, Baihui Li, Bailu Zhang, Yan Guo, Lili Yang

**Affiliations:** 1 Department of Immunology, Tianjin Medical University Cancer Institute and Hospital, Tianjin, China; 2 National Clinical Research Center for Cancer, Tianjin, China; 3 Key Laboratory of Cancer Immunology and Biotherapy, Tianjin, China; 4 Key Laboratory of Cancer Prevention and Therapy, Tianjin, China; 5 Tianjin’s Clinical Research Center for Cancer, Tianjin, China

**Keywords:** Non-small-cell lung cancer, Oncogenes

## Abstract

The protein methyltransferase SET and MYND domain-containing protein 2 (SMYD2) is a transcriptional regulator that methylates histones and nonhistone proteins. As an oncogene, SMYD2 has been investigated in numerous types of cancer. However, its involvement in lung cancer remains elusive. The prognostic value of SMYD2 expression in lung adenocarcinoma (LUAD) was determined through bioinformatics analysis, reverse-transcription polymerase chain reaction, western blotting, and immunohistochemistry. The effect of SMYD2 on LUAD cell proliferation and metastasis was explored in vivo and in vitro, and the underlying mechanisms were investigated via RNA-seq, and chromatin immunoprecipitation-quantitative PCR. SMYD2 expression was significantly upregulated in LUAD cell lines and tissues. High SMYD2 expression was associated with shorter overall and disease-free survival in LUAD patients. Inhibition of SMYD2 with SMYD2 knockdown or AZ505 dramatically inhibited the proliferation, migration, and invasion ability of GLC-82 and SPC-A1 cells and remarkably reduced tumor growth in mice. Mechanically, SMYD2 may activate the transcription of ribosomal small subunit protein 7 (RPS7) by binding to its promoter. Following overexpression of SMYD2, the proliferation, migration, and invasion of cells increased, which was partially reversed by RPS7. Thus, SMYD2 might modulate tumorigenesis and metastasis mediated by RPS7 LUAD. SMYD2 might be a prognostic biomarker and therapeutic target in LUAD.

## Introduction

Chromatin remodeling and gene regulation can be affected by various post-translational modifications (PTMs)^1,2^. Common PTMs include methylation, phosphorylation, glycosylation, ubiquitination, acetylation, and oxidation. Among these, methylation and acetylation play a crucial role in transcription and are known to be associated with tumorigenesis^3^. Several protein methyltransferases (PMTs) are involved in diverse biological processes through the epigenetic regulation of gene expression and have been implicated in various diseases. PMTs consist of two classes: protein lysine methyltransferases (PKMTs) and protein arginine methyltransferases (PRMTs), both of which are able to methylate histones and nonhistone protein substrates^4,5^. PKMTs have a vital effect on gene expression^4,6^. Generally, methylation of histone H3 lysine 9 (H3K9) and H3K27 can induce transcriptional repression, while methylation of H3K4 and H3K36 is correlated with gene activation^7^. One of the major PKMT families is the SET and MYND domain-containing (SMYD) family, which includes five members mainly located in the cytoplasm and nucleus. The overall structure of SMYD1–5 comprises the S-sequence, MYND domain, core SET domain, post-SET domain, and tetratrico-peptide repeat domain^8^.

Overexpression of SMYD2 is associated with poor prognosis for patients with cancer, such as gastric, bladder, colon, and breast cancers (BCs), along with esophageal squamous-cell carcinoma, hepatocellular carcinoma, and acute lymphoblastic leukemia^9–13^. SMYD2 is one of the most extensively studied lysine methyltransferases, which can methylate histones H3K36 and H3K4, as well as nonhistone targets^14^. SMYD2 was first reported to repress gene expression via methylation of H3K36 and through its association with Sin3A^15^. On the other hand, a subsequent study showed that methylation of H3K4 results in upregulated gene expression^14^. However, the exact mechanism underlying SMYD2-regulated histone methylation remains unclear. Furthermore, SMYD2 directly methylates estrogen receptor alpha at K266 in BC leading to its target gene activation^16^. In addition, SMYD2 promotes PTEN methylation at lysine 313 and reduces PTEN phosphorylation at serine 380, resulting in activation of the phosphatidylinositol 3-kinase-AKT pathway, thereby promoting BC cell growth^17^. Further, SMYD2 methylates retinoblastoma (RB) gene at K860, during cell cycle progression, cellular differentiation, and in response to DNA damage and at K810^18^, which leads to an increase in RB phosphorylation at serine 807/811 and promotes bladder cancer cell growth^13^. Moreover, SMYD2 promotes the growth of lung cancer via mediating ALK methylation^19^. Although several studies have reported the role and function of SMYD2 in numerous tumors, its involvement in lung adenocarcinoma (LUAD) progression has not yet been defined.

In this study, to elucidate the involvement of SMYD2 in LUAD tumorigenesis and metastasis, we evaluated the expression of SMYD2 and determined its prognostic value. Furthermore, we explored the role of SMYD2 in cell proliferation, migration, and invasion of lung cancer cells both in vitro and in xenograft mouse models, and investigated the molecular mechanisms underlying its role. This study provides strong evidence of the role of SMYD2 in LUAD tumorigenesis and metastasis. Moreover, our results suggest that SMYD2 might serve as a promising prognostic biomarker and target for LUAD therapy.

## Materials and methods

### Cell culture and reagents

All cell lines used in this study were obtained from ATCC (Manassas, VA, USA) and cultured according to the recommended protocol^20^. Normal bronchial epithelial cells BEAS-2B were cultured in a specialized medium (KCBM006, Kunming Institute of Zoology, CAS, Yunnan, China). Lung carcinoma cell lines LTEP-A2, GLC-82, A549, NCI-H460, NCI-H520, NCI-H1299, and SPC-A1 were cultured in RPMI-1640 with 10% fetal bovine serum (FBS). All cell lines were maintained in an incubator at 37 °C under 5% CO_2_ conditions. Small interfering RNAs (siRNAs) were transfected using the Lipofectamine 3000 or Lipofectamine® RNAiMAX Reagent (Invitrogen, Carlsbad, CA, USA), according to the manufacturer’s instructions. Cells seeded at a density of 5 × 10^5^ in 6-well dishes were transfected with either 20 nM siNC (control) or 20 nM specific siRNA, as per the manufacturer’s protocol (Invitrogen) for 48 h. siRNA and 3×FLAG-SMYD2 plasmid were purchased from Sangon Biotech (Shanghai, China), and SMYD2 knockdown lentivirus with luciferase from GeneChem (Shanghai, China). The siRNA and short hairpin RNA (shRNA) sequence are listed in Additional file 1: Table S1. SPC-A1 and GLC-82 cell lines with stable SMYD2 knockdown were generated according to the manufacturer’s protocol. The following antibodies were used: anti-SMYD2 antibody (immunohistochemical (IHC) staining, 1:800 dilution; immunoblotting (IB), 1:1000 dilution) from Abcam (ab195365, MA, USA), anti-ribosomal small subunit protein 7 (RPS7) antibody (IHC, 1:200 dilution) from Proteintech (14491-1-AP, Hong Kong, China), and anti-GAPDH antibody (IB, 1:1000 dilution) from Cell Signaling Technology (5174, Hong Kong, China). The SMYD2 inhibitor AZ505 was purchased from MedChem Express (Princeton, NJ, USA) and dissolved in dimethyl sulfoxide (DMSO) at a stock concentration of 20 mM. Based on the calculated IC50 value, the used concentration is 20 μM in functional experiments (Fig. S1).

### Online database analysis

The analysis of SMYD2 messenger RNA (mRNA) levels in LUAD was performed using data from the Oncomine database (https://www.oncomine.org)^21^. The filter indices were set as differential analysis (cancer versus normal analysis), cancer type (lung cancer), type (mRNA), and gene (SMYD2). The differential expression of SMYD2 between cancer specimens and normal tissues was presented using box plots. The Gene Expression Profiling Interactive Analysis (GEPIA, http://gepia.cancer-pku.cn)^22^ and PrognoScan database (http://www.abren.net/PrognoScan/)^23^ were employed for exploring SMYD2 prognosis in LUAD patients. We analyzed the prognostic value of SMYD2 and target genes using PROGgeneV2 database (http://watson.compbio.iupui.edu)^24^. Through the cBioPortal web (http://www.cbioportal.org/)^25^, we also evaluated the mutations and copy number alterations (CNAs) of the SMYD2 gene in LUAD. Somatic CNAs from RNA-sequencing (RNA-seq) data were calculated using the GISTIC (genomic identification of significant targets in cancer) algorithm. The mRNA expression data were obtained through the cBioPortal (Additional file 2: Table S2).

### Quantitative reverse-transcription polymerase chain reaction (RT-qPCR)

RT-qPCR experiments were performed as previously described^20^. Briefly, the RNA was extracted using TRIzol reagent and reverse-transcribed into cDNA. We performed the relative quantitation of mRNA using the 2^−ΔΔCt^ method, using β-actin as the internal control. The primers used are listed in Additional file 3: Table S3.

### Tumor tissue samples and IHC

For this study, 18 pairs of LUAD tissue samples and 151 LUAD tissue samples were collected from patients admitted to the Tianjin Medical University Cancer Institute and Hospital (Tianjin, China), after receiving written informed consent. The study design and procedures were approved by the Ethics Committee of Tianjin Medical University Cancer Institute and Hospital. All patients with LUAD were followed up until February 2020.

IHC was performed as previously described^20^. The tissues were assessed and analyzed by two pathologists blinded to clinicopathological characteristics of the samples and a consensus was reached. In specimens defined as positive, SMYD2 was located in the nucleus and cytoplasm of tumor cells. SMYD2 and RPS7 expression levels were assessed by multiplying the staining intensity score and frequency score. The staining intensity was classified as negative, score 0; weak, score 1; moderate, score 2; and strong, score 3. The staining frequency was scored as: 0–25%, 1; 26–50%, 2; 51–75%, 3; and 76–100%, 4. Based on the cut-off value of the receiver-operating curve analysis, if the product of staining intensity score and staining frequency score was <5.5, it was classified as SMYD2low expression group, while if it was ≥5.5, as SMYD2high expression group. Consistent with the previous method, if the product was <4.5, it was classified as RPS7low expression group, while if it was ≥4.5, as RPS7high expression group.

### CCK8 assay and colony formation assay

One thousand SPC-A1 and GLC-82 cells infected with the indicated lentivirus, plasmids, and/or siRNA were seeded in 96-well plates. Cells were incubated with CCK8 for 2 h and the optical density values were then measured at a wavelength of 450 nm using a spectrophotometer. The CCK8 assay for SPC-A1 and GLC-82 cells treated with AZ505 was performed in a similar manner. A total of 1000 SPC-A1 and GLC-82 cells infected with the relevant lentivirus were plated in 10-cm dishes with 10 mL of medium and grown for 2 weeks. The colonies were fixed and stained with 0.1% crystal violet dissolved in methanol for 20 min. Colony numbers were counted and the images were then captured. For each complete experiment, three independent samples in the indicated groups were subjected to analysis, and all experiments were performed in triplicates.

### Cell invasion and migration assay

A wound closure assay was used to determine cell migration. SPC-A1 and GLC-82 cells (7 × 10^5^ cells 2 mL^−1^) were seeded in a 6-well plate, following infection with the indicated lentivirus or treatment with AZ505, plasmids, and/or siRNA. When the cells reached 90–100% confluency, wounds were created using a sterile 10-μL pipette tip and images were taken at 0 h. Then, the cells were cultured in serum-free 1640 medium and images were taken at 48 h. In the invasion assay, transwell chamber filters (Becton Dickinson) were coated with 30 µL of Matrigel. Following infection with the lentivirus or treatment with AZ505, 2 × 10^4^ SPC-A1 and GLC-82 cells in 200 μL of serum-free 1640 medium were seeded into the upper chamber. Approximately 500 μL of 1640 medium with 10% FBS was added to the lower chamber. After 24 h, the chambers were fixed and stained with 0.1% crystal violet dissolved in methanol for 20 min. Then, the cells were counted and photographed using a microscope. There were three independent samples in each complete experiment, and all experiments were performed three times.

### Tumor growth in vivo

Female BALB/c nude mice (6–8 weeks old) were purchased from SPF Biotechnology Co., Ltd. (Beijing, China). All animal experiments were approved by the Animal Care Committee of Tianjin Medical University. GLC-82 cells stably expressing shSCR or shSMYD2 lentivirus were subcutaneously injected into the right flank of mice (1 × 10^7^ mL^−1^, 0.1 mL per mouse, *n* = 5 in each group). The tumor volume per week was calculated as follows: (length × width^2^)/2. For the AZ505 treatment assay, all mice were injected with GLC-82 cells (1 × 10^7^ mL^−1^, 0.1 mL per mouse, *n* = 6), and the tumor growth was monitored. When the tumor volume reached ~30 mm^3^, mice were randomly divided into two groups: the DMSO group and the AZ505 treatment group. DMSO and AZ505 were administered intraperitoneally at 40 mg kg^−1^ per day. Tumor volumes were recorded every 2 days. Animals were euthanized via CO_2_ suffocation 2 weeks after the first administration, and then the tumors were peeled carefully and weighted.

To examine lung cancer cell metastasis, GLC-82 cells, stably expressing luciferase and shSCR/shSMYD2 lentivirus, were injected into the tail vein of BALB/c nude mice (1 × 10^7^ mL^−1^, 0.1 mL per mouse). For AZ505 treatment, all BALB/c nude mice were injected with GLC-82 cells stably expressing luciferase and shSCR lentivirus (1 × 10^7^ mL^−1^, 0.1 mL per mouse). Each mouse was intraperitoneally injected with AZ505 (40 mg kg^−1^) and the same volume of DMSO every 2 days. The mice were sacrificed at 8 weeks post injection. The bioluminescence of the neoplasia was observed using the IVIS Imaging System (Xenogen, Baltimore, MD, USA).

### Chromatin immunoprecipitation (ChIP) assay

A total of 4 × 10^6^ shSCR- and shSMYD2-transfected GLC-82 cells were prepared for ChIP assay using the SimpleChIP^®^ Plus Enzymatic Chromatin IP Kit (Magnetic Beads). According to the manufacturer’s instruction, 10 μL sample of the diluted chromatin was taken for the input sample. Equal volumes (5 μL) of anti-SMYD2, anti-H3, and normal rabbit IgG antibodies were added to the samples for IP analysis. The purified DNA was sequenced for RT-qPCR analysis. The amplification efficiency was calculated as enrichment relative to the input. Primers used for ChIP-qPCR are listed in Additional file 3: Table S3.

### Statistical analysis

Statistical analysis was performed using GraphPad Prism 8.0.2. All data are shown as the mean ± standard deviation (SD). *P* values were calculated by two-tailed unpaired *T* test, one-way analysis of variance (ANOVA), and two-way ANOVA tests. The *χ*^2^ test was applied to evaluate the association between the expression of SMYD2 and clinicopathological characteristics of LUAD patients. Kaplan–Meier survival analysis was employed to estimate the survival rates. The univariate and multivariate analyses were also performed by SPSS 24. *P* < 0.05 was considered to denote statistical significance.

## Results

### Online database analysis shows that SMYD2 overexpression is associated with poor prognosis in patients with LUAD

**Fig. 1:**
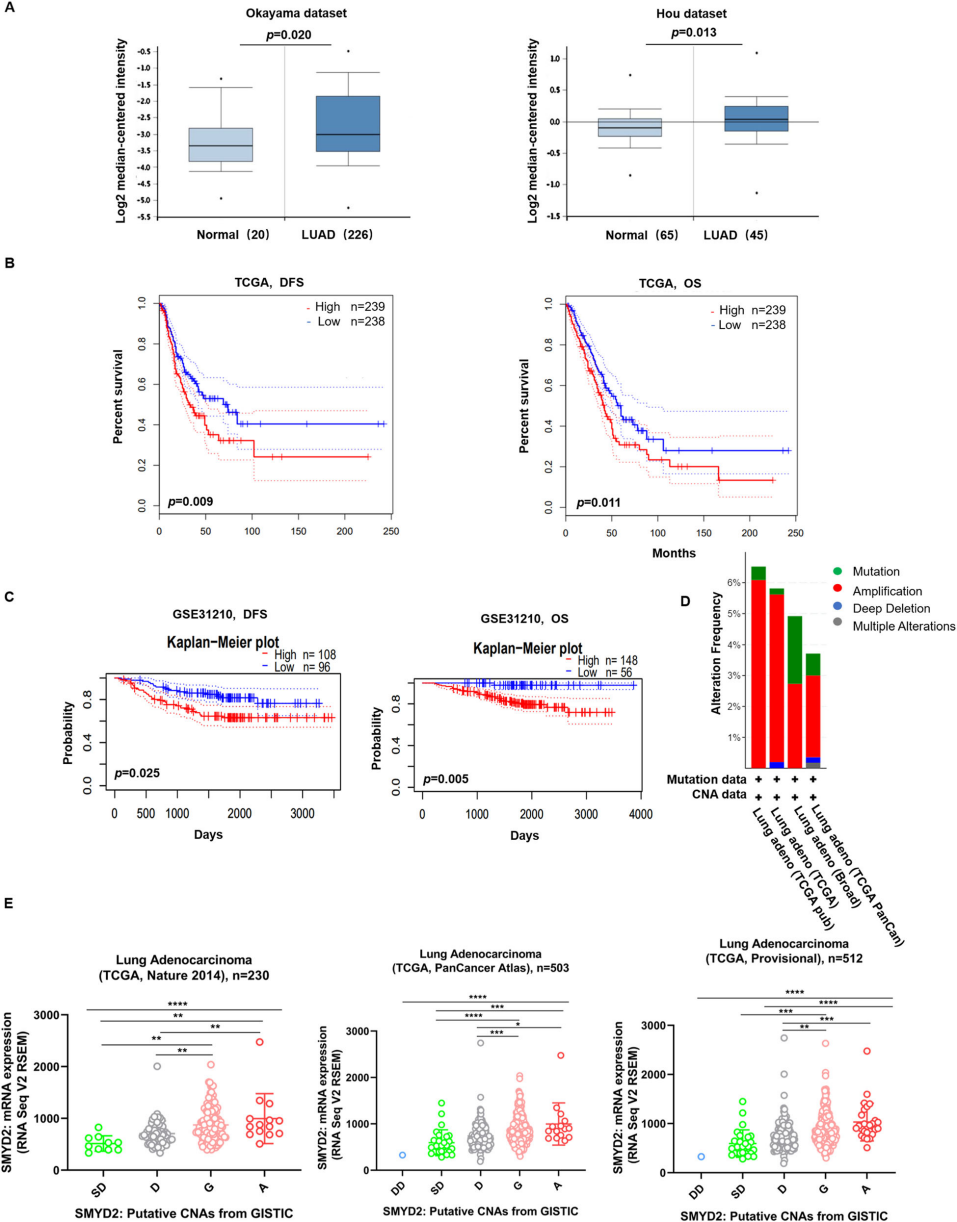
SMYD2 expression is upregulated and its overexpression is associated with poor prognosis in patients with LUAD from the online database. **A** Box plots demonstrated SMYD2 mRNA upregulation in LUAD relative to normal lung tissues (data downloaded from Oncomine). Relative SMYD2 mRNA expression in Okayama’s microarray datasets, which include 226 cases of LUAD and 20 normal lung tissues. Relative SMYD2 mRNA expression in Hou’s microarray datasets, which consist of 45 cases of LUAD and 65 normal lung tissues. **B** High SMYD2 expression levels were associated with a poorer prognosis of DFS and OS via GEPIA. **C** There was a worse prognosis in patients with overexpression SMYD2 in the LUAD cohort (GSE31210, *n* = 204). OS overall survival, DFS disease-free survival. **D** Frequency of mutation and genomic alterations in SMYD2 in LUAD was presented as a bar diagram. **E** The graph depicted the correlation between SMYD2 expression and copy number alterations in LUAD of TCGA data. Abbreviations represent the types of copy number alterations: deep deletions (DD), shallow deletion (SD), diploid (D), gain (G), and amplification (A) (*p < 0.05; ** *p* < 0.01; *** *p* < 0.001; **** *p* < 0.0001).

To determine the expression status of SMYD2 in LUAD, we analyzed its mRNA expression in LUAD and normal lung tissues using online databases. We performed data mining and analyzed SMYD2 transcriptional profiles from the publicly available Oncomine datasets. Data from Okayama’s microarray datasets (226 LUADs and 20 normal lung tissues)^26^ and Hou’s microarray datasets (45 LUADs and 65 normal tissues)^26^ showed significantly higher mRNA expression of SMYD2 in LUAD than in normal lung tissues (*p* = 0.020 and *p* = 0.013, respectively, Fig. 1A).

Next, we evaluated whether SMYD2 expression had an effect on the survival rates of cancer patients. We retrieved the survival curves of LUAD tissues with high (*n* = 239) and low (*n* = 238) expression of SMYD2 from the GEPIA database. High SMYD2 expression levels were associated with a poorer prognosis of disease-free survival (DFS) and overall survival (OS) (*p* = 0.009 and *p* = 0.011, respectively, Fig. 1B) than low SMYD2 expression levels. In addition to the RNA-seq data in The Cancer Genome Atlas (TCGA) of SMYD2 via GEPIA, we also performed microarray analysis to examine the prognostic potential of SMYD2 in LUAD using the PrognoScan database. One cohort (GSE31210) included 204 samples with different stages of LUAD. The results indicated a worse prognosis in patients with overexpression of SMYD2 than in those without SMYD2 overexpression (DFS *p* = 0.025; OS *p* = 0.005) (Fig. 1C). Analysis of the relationship between the expression profile of SMYD2 and the clinicopathological characteristics of LUAD patients from the TCGA and GEO databases showed no obvious difference between the high- and low-SMYD2 groups in age, gender, smoking status, tumor node metastasis (TNM) stage, T stage, and N stage (Tables S4–S6). Therefore, these results suggest that elevated SMYD2 expression is a risk factor in LUAD and can be considered a potential prognostic biomarker for patients with LUAD.

DNA CNAs are correlated with gene dysregulation in various cancers^27^. We speculated that an increase in CNAs may lead to SMYD2 gene overexpression in LUAD. We, therefore, investigated the mutations and CNAs in SMYD2 in a cohort of patients with LUAD using the cBioPortal website. The mutation frequencies were only ~2% and 0.2% in the broad and TCGA datasets, respectively. CNA was the dominant alteration, and the amplification frequencies were ~4–6% (Fig. 1D). There was a significant correlation between CNAs and the mRNA expression level of SMYD2 in the data obtained from TCGA database (one-way ANOVA analysis, *p* = 0.0001) (Fig. 1E). Therefore, a gain in CNAs might lead to SMYD2 mRNA overexpression.

### Validation of SMYD2 overexpression and its association with poor prognosis in LUAD via in vitro experiments

**Fig. 2:**
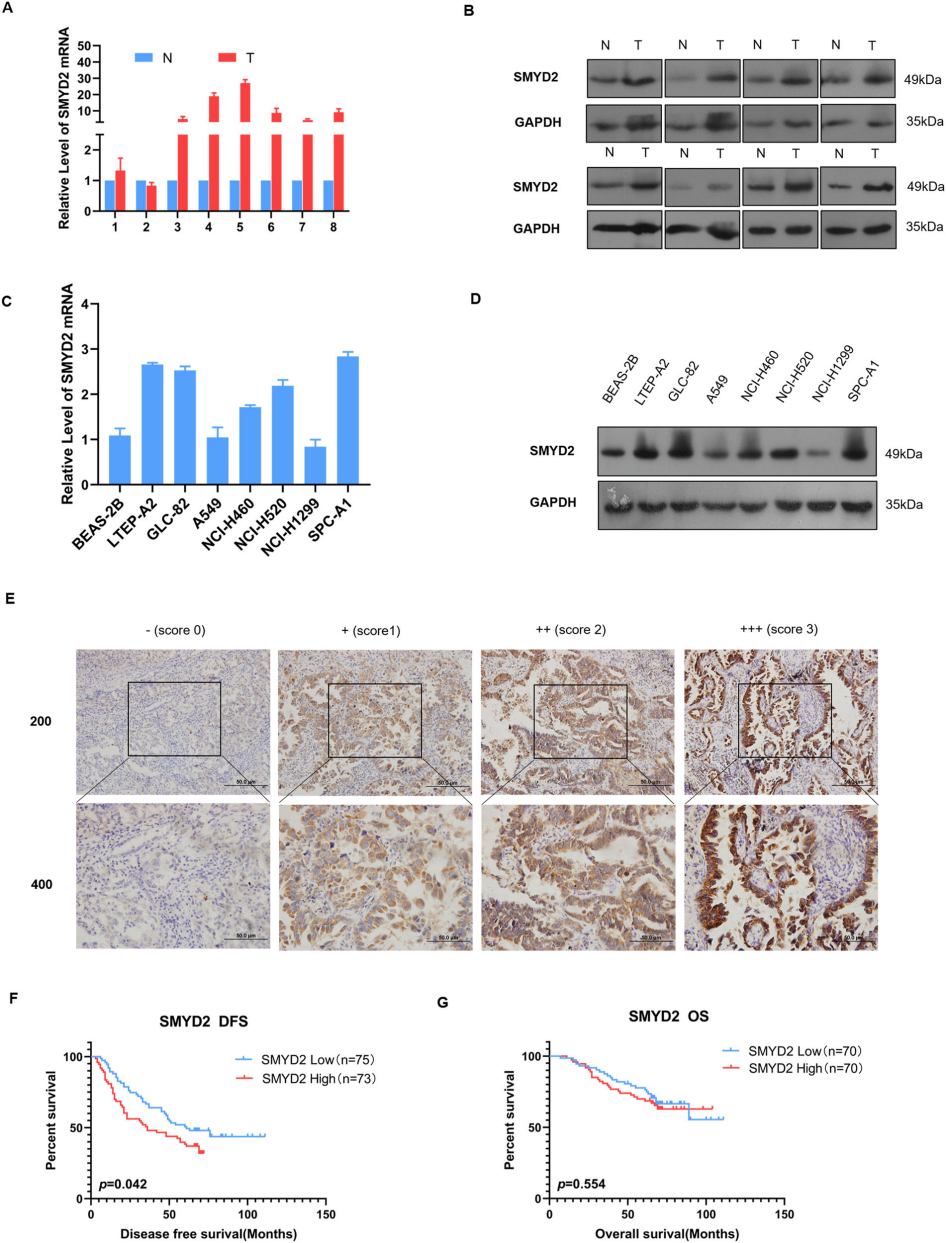
Upregulated expression of SMYD2 and its poor prognosis value was validated via in vitro experiment. **A**, **B** The SMYD2 mRNA and protein level was significantly upregulated in surgically removed tumor tissues (T), compared to the adjacent normal lung tissues (N) by RT-qPCR and western blotting. **C**, **D** The mRNA and protein expression of SMYD2 in lung cancer cell lines was higher than human bronchial epithelial cell line (BEAS-2B). **E** SMYD2 protein expressions were observed by immunohistochemical staining in 151 cases of LUAD samples. Scores 0, 1, 2, and 3 represent negative (–), weak positive (+), moderate positive (++), and strong positive (+++) expression, respectively. Original magnification: ×200 and ×400. Scale bars, 50 μm. **F**, **G** The Kaplan–Meier survival analysis of DFS and OS between low and high SMYD2 expression groups.

To verify the results obtained from the online bioinformatics datasets, we firstly evaluated SMYD2 expression in eight pairs of LUAD and peritumor specimens via RT-qPCR and western blotting. Consistent with the results from the public database, SMYD2 mRNA levels in six out of eight samples were significantly higher in LUAD tumor tissues than in the paired adjacent normal tissues (Fig. 2A, B). Similarly, an increasing trend in SMYD2 protein expression was observed in the LUAD tissues. Subsequently, the expression spectrum of SMYD2 was analyzed in LTEP-A2, GLC-82, A549, NCI-H460, NCI-H520, NCI-H1299, and SPC-A1 cell lines, along with BEAS-2B cells. The mRNA and protein levels of SMYD2 were markedly higher in LTEP-A2, GLC-82, NCI-H460, NCI-H520, and SPC-A1 cell lines than in BEAS-2B cells (Fig. 2C, D). These evidences suggest that SMYD2 could play a role in the tumorigenesis of LUAD.

Next, we further assessed the significance of SMYD2 expression in 151 LUAD specimens. IHC analysis revealed that SMYD2 was abundant in both the cytoplasm and nucleus of the tumor cells (Fig. 2E). We further verified the relationship between SMYD2 expression and the clinicopathological characteristics of patients (Table 1). Consistent with the results from the public database, there was no obvious difference in age, gender, smoking status, and TNM stage between the two groups (Table 1). Further, patients with low expression of SMYD2 showed higher DFS and OS compared to those with high expression (Fig. 2F). However, there was no statistically significant difference in the OS (*p* = 0.554, Fig. 2G). In addition, the univariate analysis showed the SMYD2 expression level, TNM stage, T stage, and N stage were univariately correlated with DFS (Table 2). We then employed a multivariate analysis, which suggested that a higher SMYD2 expression level (*p* = 0.013, HR = 1.711) and TNM stage (*p* < 0.001, HR = 1.824) were related to poor prognosis (Table 2). Collectively, these results demonstrated that SMYD2 was associated with a poor prognosis of LUAD patients.

**Table 1:** Correlation analysis between SMYD2 level and clinical–pathological characteristics of LUAD patients.

Characteristics	*n*	SMYD2		*χ*^2^	*p* Value
		High (%)	Low (%)		
T stage				4.621	0.204
T1	87	47 (54.0)	40 (46.0)		
T2	51	20 (39.2)	31 (60.8)		
T3	8	4 (50.0)	4 (50.0)		
T4	5	4 (80.0)	1 (20.0)		
N stage				0.885	0.707
N0	93	49 (52.7)	44 (47.3)		
N1	11	5 (45.5)	6 (54.5)		
N2	47	21 (44.7)	26 (55.3)		
TNM stages				0.090	0.975
I	77	39 (50.6)	38 (49.4)		
II	26	13 (50.0)	13 (50.0)		
III	48	23 (47.9)	25 (52.1)		
Smoking index				0.490	0.484
<400	100	51 (51.0)	49 (49.0)		
≥400	49	22 (44.9)	27 (55.1)		
Age (years)				0.069	0.793
≤60	89	45 (50.6)	44 (49.4)		
>60	62	30 (48.4)	32 (51.6)		
Gender				1.128	0.288
Males	68	31 (45.6)	37 (54.4)		
Females	83	44 (53.0)	37 (44.6)

**Table 2:** Univariate and multivariate analysis between clinical and survival in patients with lung adenocarcinoma.

Characteristics	*n* (%)	Univariate analysis		Multivariate analysis	
		Median DFS	*p* Value	DFS HR (95% CI)	*p* Value
T			**0.004**		
T1	87 (57.6)	69			
T2	51 (33.8)	21			
T3	8 (5.3)	19			
T4	5 (3.3)	57			
N			**0.000**		
N0	93 (61.6)				
N1	11 (7.3)	57			
N2	47 (31.1)	23			
TNM			**0.000**	1.824 (1.445–2.301)	**0.000**
I	77 (51.0)				
II	26 (17.2)	37			
III	48 (31.8)	24			
Smoking index			0.058		
<400	100 (67.1)	45			
≥400	49 (32.8)				
Age (years)			0.984		
≤60	89 (58.9)	49			
>60	62 (41.1)	48			
Gender			0.250		
Males	68 (45)	60			
Females	83 (55)	47			
SMYD2			**0.042**	1.711 (1.118–2.618)	**0.013**
Low	76 (50.3)	61			
High	75 (49.7)	36			

Bold values indicate statistical significance *p* < 0.05.

### Knockdown of SMYD2 results in decreased tumor growth and *metastasis* in vitro and in vivo

**Fig. 3:**
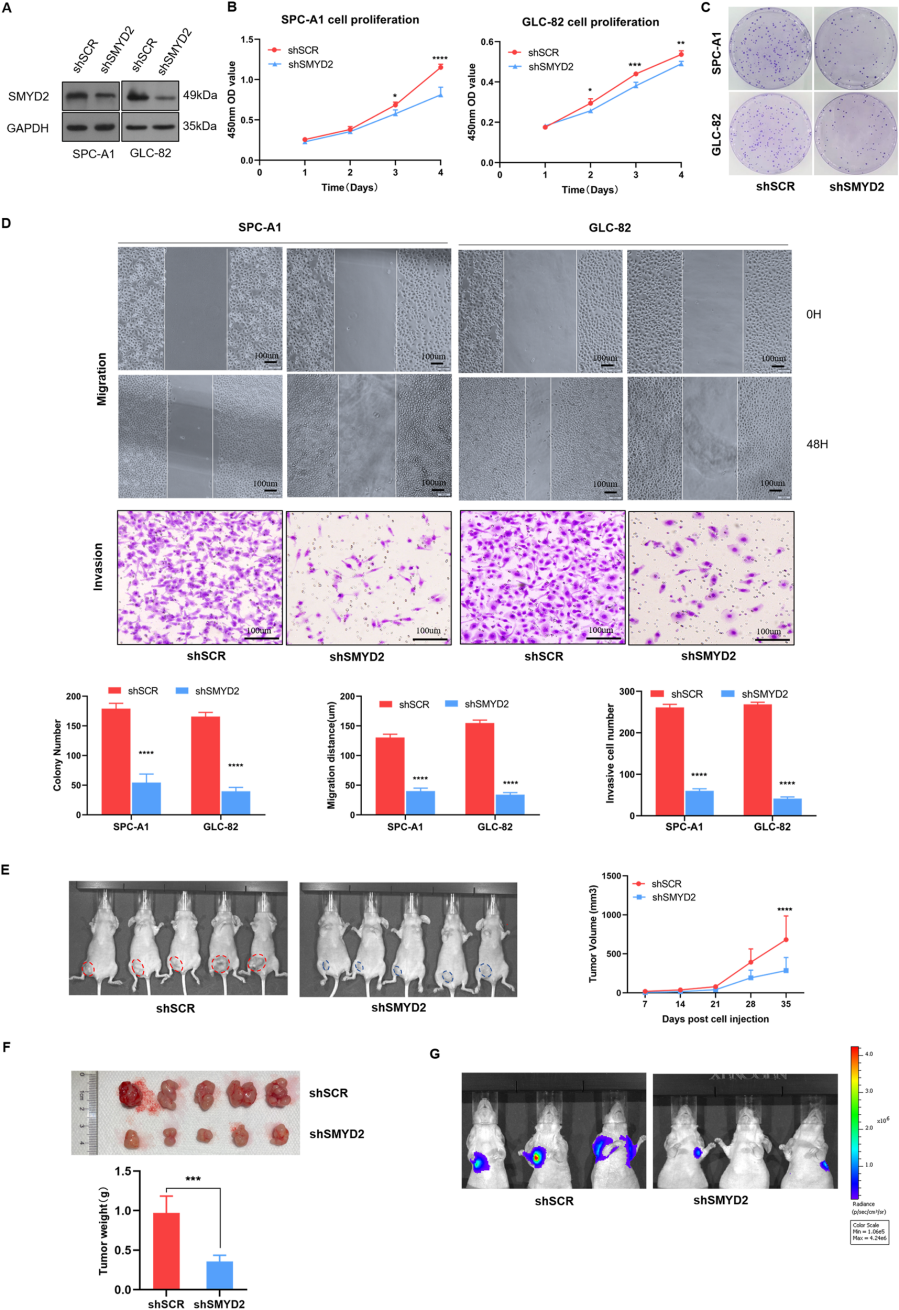
Knockdown of SMYD2 results in a decrease in tumor growth and metastasis in vitro and in vivo. **A** Knockdown efficiency of SMYD2 in SPC-A1 and GLC-82 cells were confirmed through western blotting, with GAPDH as an internal control. **B**, **C** SMYD2 knockdown inhibited both SPC-A1 and GLC-82 cells proliferation as detected by CCK8 proliferation assay and colony formation assay. *p < 0.05; **p < 0.01; ***p < 0.001; ****p < 0.0001 compared with the control from two-way ANOVA comparisons test. **D** SMYD2 knockdown markedly attenuated cell migration and invasion in SPC-A1 and GLC-82 cells measured by wound healing and Transwell assays. The images represent one field under the microscope (×200 magnification). ****p < 0.0001 compared with unpaired *T* test. Scale bars, 100 μm. **E**, **F** The in vivo xenograft model showed that SMYD2 knockdown (shSMYD2) reduced tumor volume and weight compared with the shSCR control group. **G** The vivo metastatic model showed that SMYD2 knockdown group could reduce the metastatic lesion than those in the control group. Two-way ANOVA comparisons test was performed on tumor volume. Unpaired *T* test was performed on tumor weight, ***p < 0.001, ****p < 0.0001.

To explore and verify whether SMYD2 is a potential oncogene in LUAD, we performed cell proliferation, migration, and invasion assays. First, SPC-A1 and GLC-82 cells were infected with SMYD2-specific shRNA and the corresponding negative control. Western blot analysis revealed that shSMYD2-infected cells had dramatically lower protein levels of SMYD2 compared to the control-transfected cells (Fig. 3A). Interestingly, we observed a significant decrease in the proliferative ability of SMYD2-silenced cells as detected by the CCK8 assay (Fig. 3B). Consistent with the CCK8 data, colony formation by SPC-A1/shSMYD2 and GLC-82/shSMYD2 cells was notably inhibited (Fig. 3C). Next, we sought to determine the potential effect of SMYD2 in tumor metastasis. Migration and invasion assays showed that SMYD2 knockdown markedly attenuated the migration and invasion ability of SPC-A1 and GLC-82 cells (Fig. 3D). Furthermore, we generated a xenograft model to investigate the biological function of SMYD2 in vivo. As shown in Fig. 3E,F, SMYD2 knockdown resulted in markedly lower tumor volume and weight compared to the control. To explore the role of SMYD2 in cancer metastasis, we used a tail vein injection mouse model and detected lung metastasis through IVIS. We found that SMYD2 knockdown caused a relatively more reduction of the metastatic lesion compared to the control group (Fig. 3G). These results provide evidence that SMYD2 acts as a tumor activator for the growth and metastasis of LUAD cells.

### Inhibition of SMYD2 with AZ505 suppresses tumor growth in vitro and in vivo

**Fig. 4:**
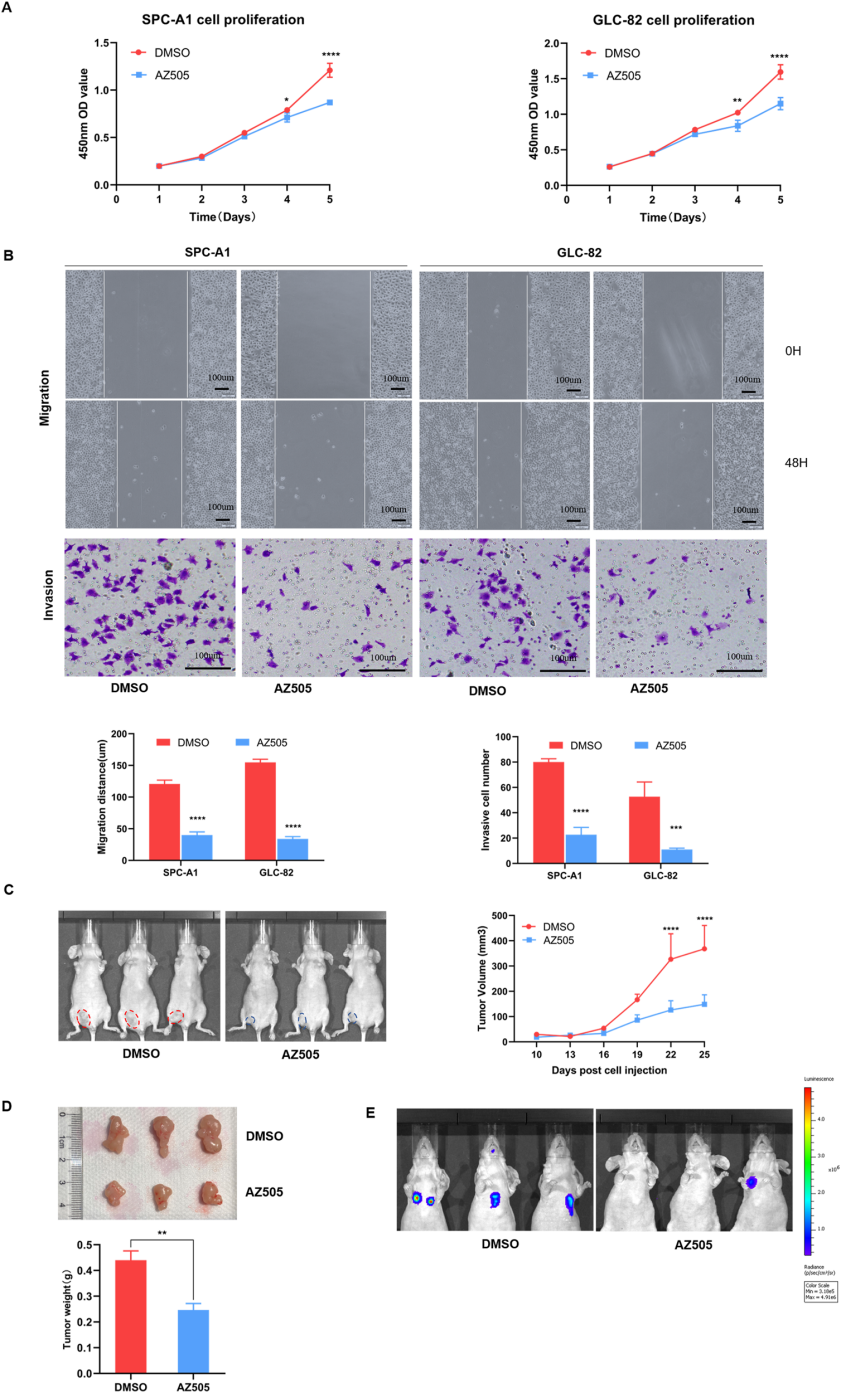
Inhibition of SMYD2 with AZ505 suppresses tumor growth in vitro and in vivo. **A** AZ505 treatment (20 μM, 24 h) inhibited SPC-A1 and GLC-82 cells proliferation, as examined by CCK8 proliferation assay. *p < 0.05; **p < 0.01; ***p < 0.001; ****p < 0.0001 compared with the control from two-way ANOVA comparisons test. **B** Migration (would healing) and invasion (Transwell) assays indicated that AZ505 (20 μM, 24 h) treatment suppressed SPC-A1 and GLC-82 cell migration and invasion. Representative photographs were taken at ×200 magnification. ****p < 0.0001 compared with unpaired *T* test. Scale bars, 100 μm. **C**, **D** The in vivo xenograft model showed that SMYD2 AZ505 treatment reduced tumor volume and weight compared with the DMSO group. **E** The in vivo metastasis model showed that the metastatic lesion was significantly suppressed by the intraperitoneal injection of AZ505 compared to those in the DMSO group. Two-way ANOVA comparisons test was performed on tumor volume. Unpaired *T* test was performed on tumor weight, ***p < 0.001, ****p < 0.0001.

AZ505 is regarded as an effective and specific inhibitor of SMYD2, which competitively inhibits the binding of other substrates by occupying the peptide-binding groove of the enzyme. Following treatment with AZ505, the proliferation of LUAD cell lines was significantly inhibited as examined by CCK8 analysis (Fig. 4A). Wound healing and transwell assays demonstrated that SPC-A1 and GLC-82 cells treated with AZ505 had a lower migration and invasion ability compared to DMSO-treated cells (Fig. 4B). Using our xenograft model, daily intraperitoneal injections of AZ505 at a dose of 40 mg per kg of body weight led to reducing tumor growth (Fig. 4C) and tumor weight (Fig. 4D). By observing lung tissue surfaces in the BALB/c nude mouse metastasis model, the metastatic lesion was significantly suppressed by the intraperitoneal injection of AZ505 compared to those in the DMSO group (Fig. 4E). These results suggest that SMYD2 may serve as a potential target for the treatment of LUAD.

### Expression of the RPS7 is positively correlated with the expression of SMYD2 and LUAD tumorigenesis

**Fig. 5:**
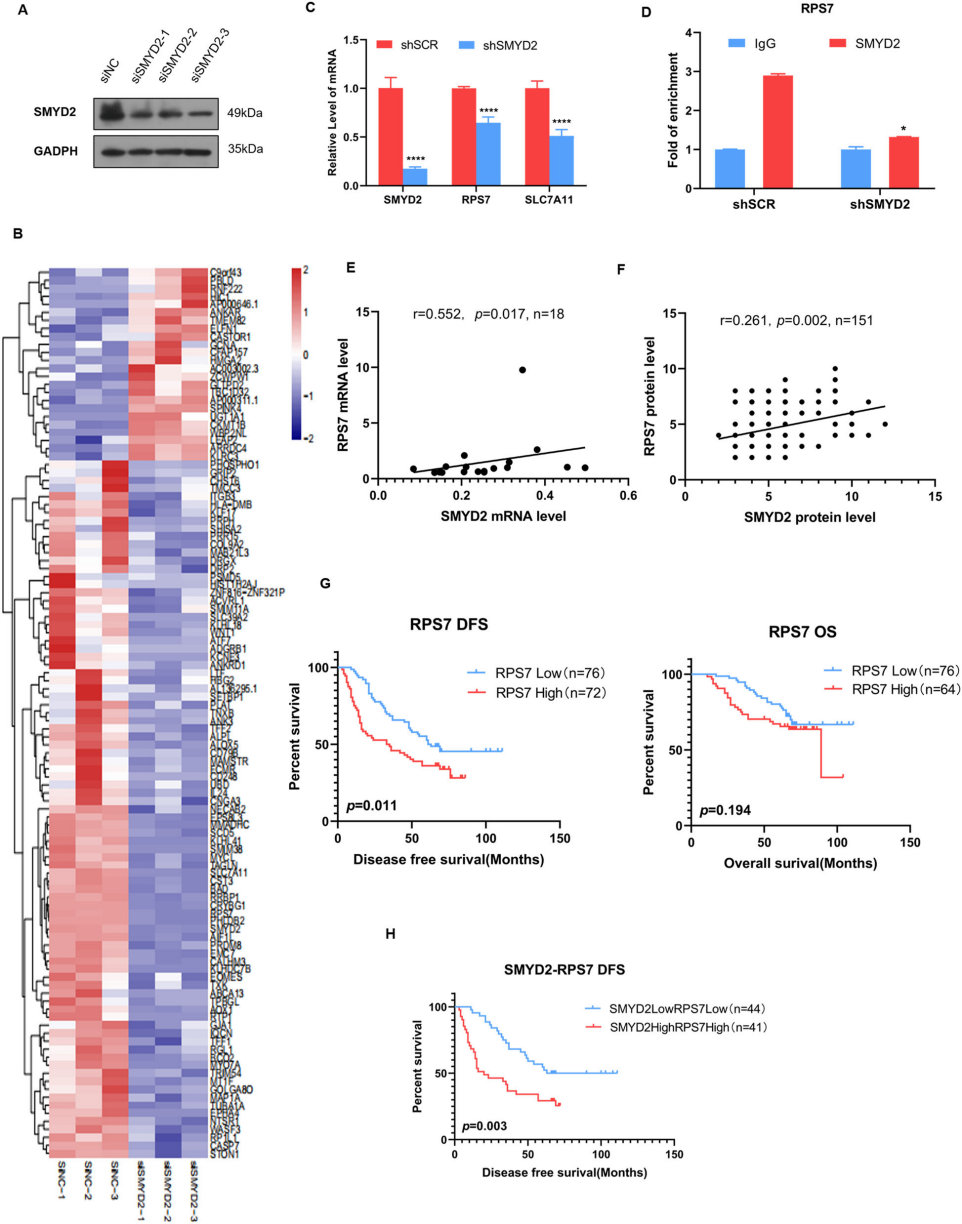
Expression of RPS7 is positively correlated with the expression of SMYD2 and LUAD tumorigenesis. **A** Knockdown efficiency of SMYD2 in GLC-82 cells was confirmed through western blotting, with GAPDH as an internal control. **B** The heat map showed that the knockdown of SMYD2 affected 111 genes expression, including 24 upregulated and 87 downregulated genes. The samples were from three independent transfections with three different siRNA sequences. **C** The mRNA expression of the indicated genes in control or SMYD2 knockdown GLC-82 cells. The error bars represent the mean ± SD of three independent experiments. ****p < 0.0001 (two-tailed unpaired *T* test). **D** ChIP-qPCR assays indicated that loss of SMYD2 diminished the enrichment on the promoter of the *RPS7* gene. *p < 0.05 (two-tailed unpaired *T* test). **E** There was a positive correlation between the expression of SMYD2 and RPS7 mRNA expression by RT-qPCR using 18 lung adenocarcinoma tissue samples (*r* = 0.552, *p* = 0.017, Spearman’s correlation analysis). **F** The protein expression of SMYD2 was positively correlated with RPS7 by IHC analysis (*r* = 0.261, *p* = 0.002, Spearman’s correlation analysis). **G** The patients with low expression of RPS7 showed higher DFS (*p* = 0.011) and OS (*p* = 0.194) compared to those with high expression. **H** The patients with SMYD2high/RPS7high had worse survival outcome, compared with SMYD2low/RPS7low patients from our hospital (*p* = 0.003).

SMYD2 overexpression in lung cancer tissues and cell lines may influence the expression of a variety of genes. To elucidate the underlying mechanisms and identify specific target genes of SMYD2, we knocked down SMYD2 expression in GLC-82 cells using specific siRNA and verified the knockdown efficiency via western blotting (Fig. 5A). Sequencing results showed that the knockdown of SMYD2 affected the expression of 111 genes, including 24 upregulated and 87 downregulated genes (Fig. 5B), indicating that the reduction in SMYD2 levels led to transcriptome alteration. Pathway enrichment analysis illustrated that knockdown of SMYD2 might influence several signaling pathways, including the apoptotic pathway, focal adhesion pathway, and ECM–receptor interaction pathway (Fig. S2A), which are all related to tumorigenesis and tumor progression.

Among the altered genes, we identified RPS7^28^ and SLC7A11^29^, which were upregulated by the TCGA dataset analysis in LUAD (Fig. S3A, B) and reported to participate in tumorigenesis. Further, RT-qPCR demonstrated that RPS7 and SLC7A11 were significantly downregulated in SMYD2-silenced GLC-82 cells (Fig. 5C). Further, we assessed the relationship between SMYD2 and RPS7 through Spearman’s correlation analysis using the TCGA database. As shown in Fig. S2B, C, there was a positive correlation between the expression of SMYD2 and that of RPS7 (*r* = 0.12, *p* = 0.0064) and SLC7A11 (*r* = 0.17, *p* < 0.001). To explore the mechanism underlying its involvement in lung cancer, we analyzed the public database. The groups were divided into the high and low groups according to the median of RPS7 and SLC7A11 expression. We found that the patients with overexpressed RPS7 and SLC7A11 had significantly worse OS than those without (Fig. S3C, D). Importantly, ChIP-qPCR assays indicated that the loss of SMYD2 diminished the enrichment of SMYD2 on the RPS7 gene promoter (Fig. 5D), whereas SMYD2 did not enrich on SLC7A11 promoter (Fig. S3E). These results implicated that SMYD2 activated the transcription of RPS7. Therefore, we mainly focused on RPS7 as a potential target gene to explore the function in subsequent experiments. In addition, we further verified the association between RPS7 and SMYD2 by RT-qPCR with 18 LUAD tissue samples. The mRNA expression of SMYD2 was positively correlated with RPS7 (*r* = 0.552, *p* = 0.017) (Fig. 5E). To further identify the relationship of the protein level, we did RPS7 staining with IHC in our 151 LUAD patients. The correlation of RPS7 expression with SMYD2 expression was analyzed. At the same time, the survival outcome of RPS7 expression was evaluated. The result showed that the protein expression of SMYD2 was positively correlated with RPS7 (*r* = 0.261, *p* = 0.002) (Fig. 5F). The patients with low expression of RPS7 showed higher DFS (*p* = 0.011) and OS (*p* = 0.194) compared to those with high expression (Fig. 5G). Finally, patients from the GSE13213 dataset were divided into two groups to determine their OS. The results demonstrated that patients with SMYD2high/RPS7high expression were associated with worse survival outcomes compared to those with SMYD2low/RPS7low expression (Fig. S2D). To verify this result, we analyze the SMYD2/RPS7 expression with IHC from our patients. The result indicated that patients with SMYD2high/RPS7high expression were associated with worse survival compared to those with SMYD2low/RPS7low expression in DFS (*p* = 0.003) (Fig. 5H). These findings indicate that the upregulated expression of SMYD2 and/or RPS7 is heavily involved in LUAD tumorigenesis.

### SMYD2 promotes LUAD carcinogenesis and metastasis mediated by RPS7

**Fig. 6:**
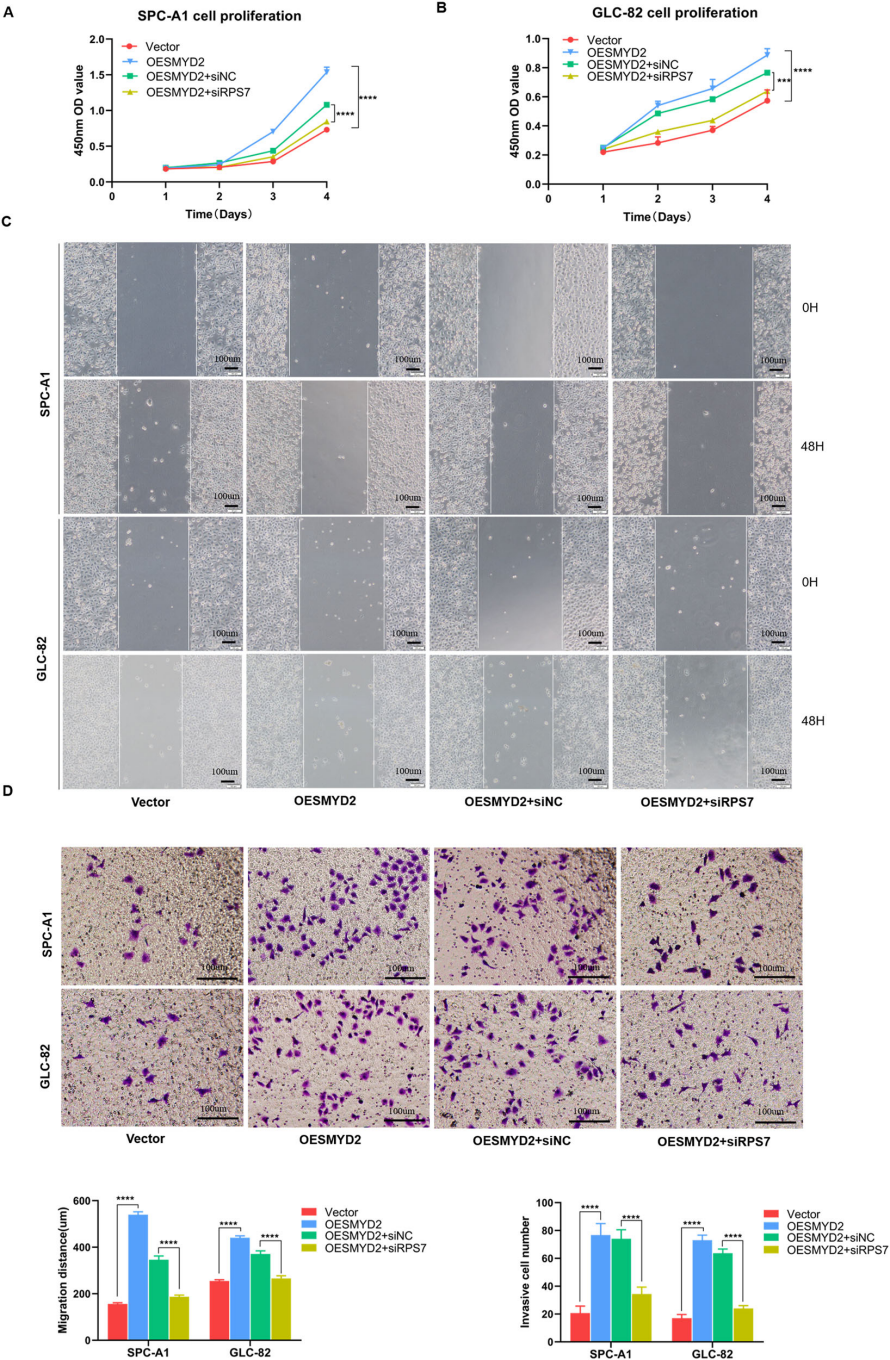
SMYD2 promotes LUAD carcinogenesis and metastasis mediated by RPS7. **A**, **B** CCK8 assay showed that RPS7 depletion partially reversed SMYD2-enhanced proliferation in SPC-A1 and GLC-82 cells. **C**, **D** RPS7 depletion attenuated the effect of SMYD2 on cell invasion and migration, as measured by wound healing and transwell assay. The images represent one field under the microscope (×200 magnification). ***p < 0.001; ****P < 0.0001 compared with unpaired *T* test. Scale bars, 100 μm.

As SMYD2 promotes tumor growth and metastasis in LUAD and activates RPS7 expression in LUAD cells, we further explored the role of RPS7 in SMYD2-regulated cell carcinogenesis and metastasis. For this purpose, SPC-A1 and GLC-82 cells were transiently transfected with vector control, 3×FLAG-SMYD2 (OESMYD2), OESMYD2, and siRPS7 or siNC control. Compared to the vector control, SPC-A1 and GLC-82 cells exposed to overexpressing SMYD2 exhibited an increase in the proliferation capacity of cells and RPS7 depletion partially reversed SMYD2-enhanced proliferation from growth curve assays (Fig. 6A, B). To investigate whether RPS7 mediates the SMYD2-regulated promotion of LUAD cell metastasis, the migration, and invasion capability of the cells shown in Fig. 6C,D was assessed. In wound closure migration assay, the results showed that SMYD2 overexpression was associated with a marked increase after 48 h of migration distances. Furthermore, in agreement with the functional link between SMYD2 and RPS7 described previously, the increase in migration distances resulting from SMYD2 overexpression was partially reversed by knockdown of RPS7. Using transwell invasion assay, we found that a significant increase in invasion abilities was associated with overexpression of SMYD2, which could be restored by combining with RPS7 depletion. Collectively, these results confirm that SMYD2 promotes the proliferation and metastasis potential possibly by activating RPS7 expression in LUAD cells.

## Discussion

The PMT SMYD2^12,14,30^, as an oncogene, is closely connected with transcriptional regulation, epigenetics, and tumorigenesis^31,32^. SMYD2 is highly expressed and related to poor prognosis in multiple cancers, including BC^11^, esophageal squamous cell carcinoma^10^, acute lymphoblastic leukemia^12^, gastric cancer^9^, hepatocellular carcinoma^33^, and colon cancer^34^. In our study, we found that SMYD2 expression was upregulated in sample datasets procured from online databases, as well as in LUAD cell lines and tissues. Consistent with the findings from previous studies, our results showed that the overexpression of SMYD2 was associated with a poor prognosis of patients with LUAD. However, there was no significant difference in the OS of patients from our hospital. Collectively, our results indicate that SMYD2 could be a promising biomarker for the diagnosis and prognosis of LUAD in patients. In addition, via a series of in vitro experiments, we also found that SMYD2 knockdown or AZ505-mediated SMYD2 inhibition could dramatically inhibit the cell proliferation, migration, and invasion ability of LUAD cells. Conversely, SMYD2 overexpression promoted the carcinogenesis and metastasis of LUAD cells. In addition, in vivo studies using mouse models revealed that SMYD2 knockdown or AZ505-mediated SMYD2 inhibition remarkably reduced tumor growth and metastasis. These data provide the basic foundation to further explore whether SMYD2 could be a new target in tumorigenesis and metastasis of LUAD.

Lysine methylation on H3K4 and H3K36 generally corresponds to gene activation^7^. In addition, several studies revealed that SMYD2 could methylate nonhistone proteins and thus further affect gene expression. Through RNA-seq, RT-qPCR, and TCGA data analysis, we identified that RPS7 and SLC7A11 might be the most likely factors involved in the proliferation and metastasis of LUAD. RPS7 is an important component of the small subunit of ribosomes, which is a crucial player in the process of protein translation^35^. The dysregulation of protein translation appears to play a pivotal role in the development of many tumors^36,37^. Upregulation of RPS7 was correlated with worse recurrence-free survival and OS in patients with prostate cancer and overexpression of RPS7 dramatically increased prostate cancer cell growth^36^. RPS7 also promotes cell migration by regulating the epithelial-to-mesenchymal transition^28^. However, not much is known about its association with lung cancer development. SLC7A11 as cystine–glutamate transporter regulates cystine–glutamate exchange. The expression of SLC7A11 was significantly higher in melanoma and lung cancer patients. The overexpression of SLC7A11 is correlated with worse survival in lung cancer^29,38^. Consistent with the results from previous studies, the analysis of online databases showed that patients with RPS7 and SLC711A overexpression had significantly worse OS in LUAD than those with low expression. Our findings also revealed that SMYD2 activated RPS7 and SLC7A11 expression. However, there was no enrichment at the SLC711A gene promoter by ChIP-qPCR assays. We speculate that SMYD2 may indirectly regulate the expression of SLC7A11. The underlying mechanisms are still unclear and worth probing. The upregulation of RPS7 may be crucial for SMYD2-mediated tumor growth and metastasis. Therefore, we mainly focused on RPS7 to explore the role of RPS7 in SMYD2-regulated cell carcinogenesis and metastasis. Through rescue experiments, we found that SMYD2-enhanced proliferation, migration, and invasion capability were partially reversed by RPS7 depletion. Collectively, this research provided a deeper insight into the potential role of SMYD2 and the possible target genes in carcinogenesis.

Interestingly, ChIP-qPCR assay results indicated that SMYD2 knockdown reduced the enrichment of the RPS7 gene promoter, further implying that SMYD2 could play a role in the transcriptional activation of RPS7. However, the detailed mechanism underlying its transcriptional regulation is still unclear. SMYD2 was previously shown to transcriptionally suppress the target genes *RASSF1*, *SIAH1*, and *AXIN2* via interactions with EZH2^39^. Moreover, a recent study showed that the downregulation of APC2 was due to SMYD2-mediated DNA methylation, which involved the recruitment of DNMT1 by SMYD2^40^. The fact that we could not validate the potential role of and the detailed molecular mechanism underlying, SMYD2-mediated RPS7 gene activation constitutes another limitation of our research. Therefore, the mechanism underlying SMYD2-mediated RPS7-driven lung cancer growth will need to be further explored in subsequent studies.

## Conclusions

In summary, SMYD2 was overexpressed, which was associated with poor prognosis in LUAD patients. SMYD2 might promote carcinogenesis and metastasis of LUAD cells by transcriptionally activating RPS7. These results suggest that SMYD2 might be a candidate biomarker of lung cancer prognosis and could be used as a potential therapeutic target in lung cancer to improve clinical outcomes.

## Supplementary information

Figure S1

Figure S2

Figure S3

Table S1

Table S2

Table S3

Table S4

Table S5

Table S6

Supplementary Figure Legends
