# Supplementary figures and images for: SMYD2 promotes tumorigenesis and metastasis of lung adenocarcinoma through RPS7
Source: Cell Death Dis. 2021 May 2;12(5):439. doi: 10.1038/s41419-021-03720-w (PMC8089105; doi:10.1038/s41419-021-03720-w)

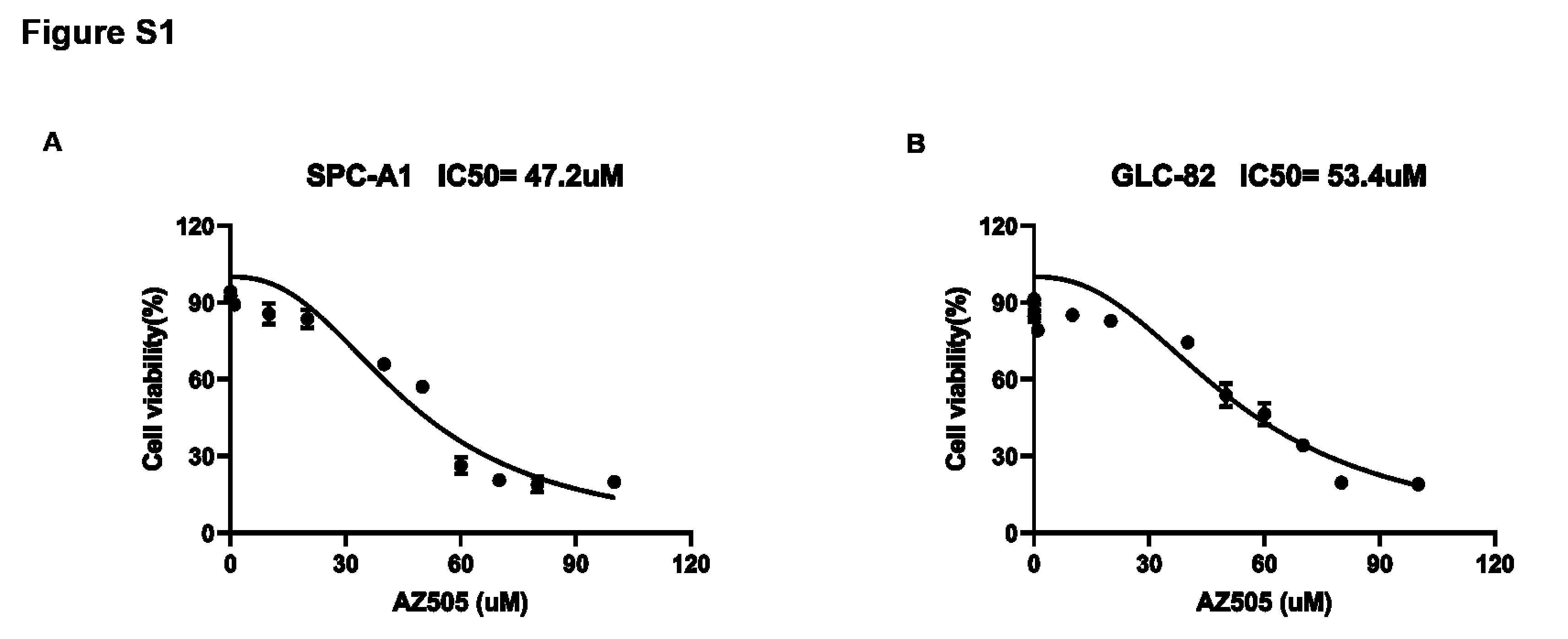

Supplement: Supplementary file 1 — Figure S1 [file 41419_2021_3720_MOESM1_ESM.png]

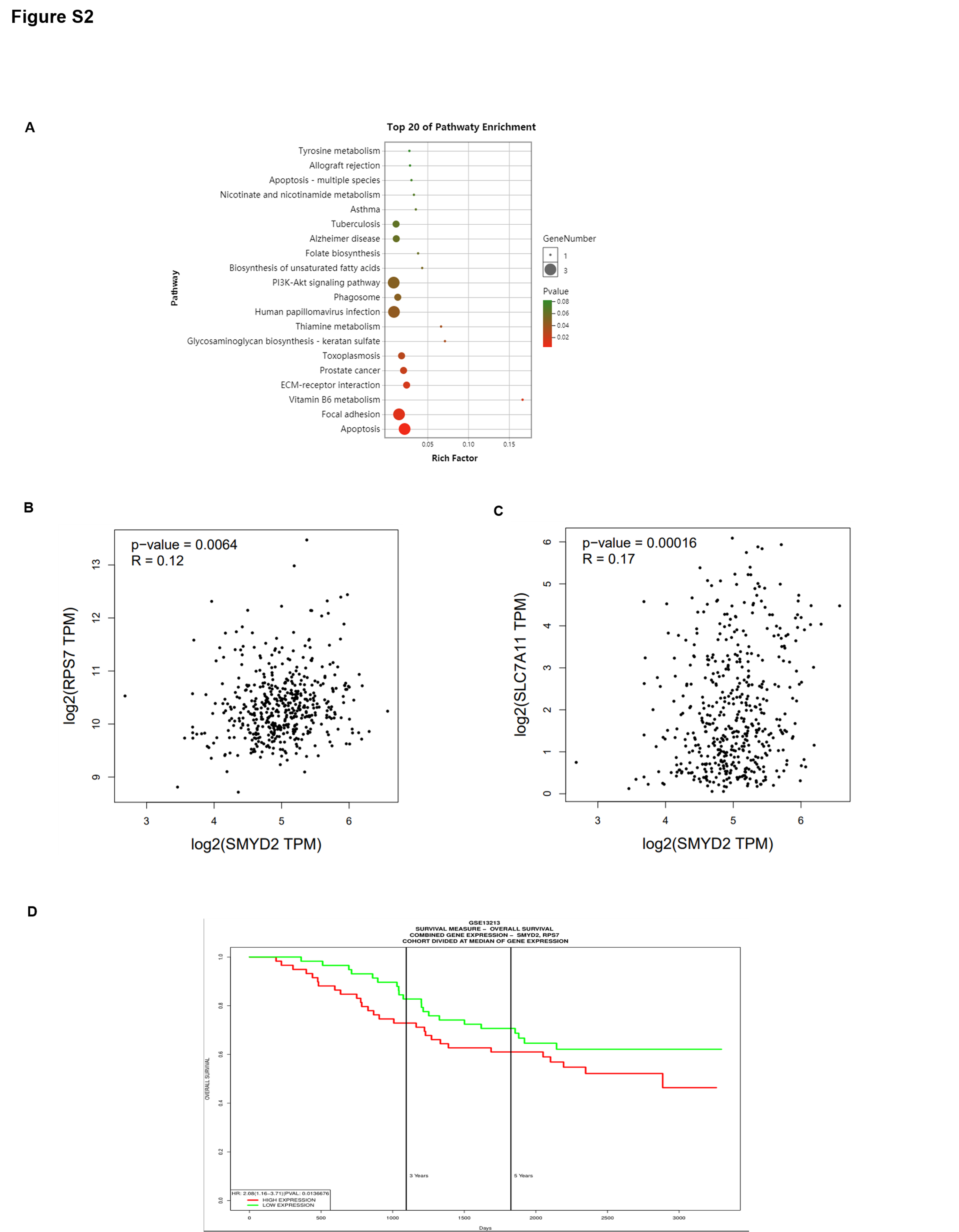

Supplement: Supplementary file 2 — Figure S2 [file 41419_2021_3720_MOESM2_ESM.png]

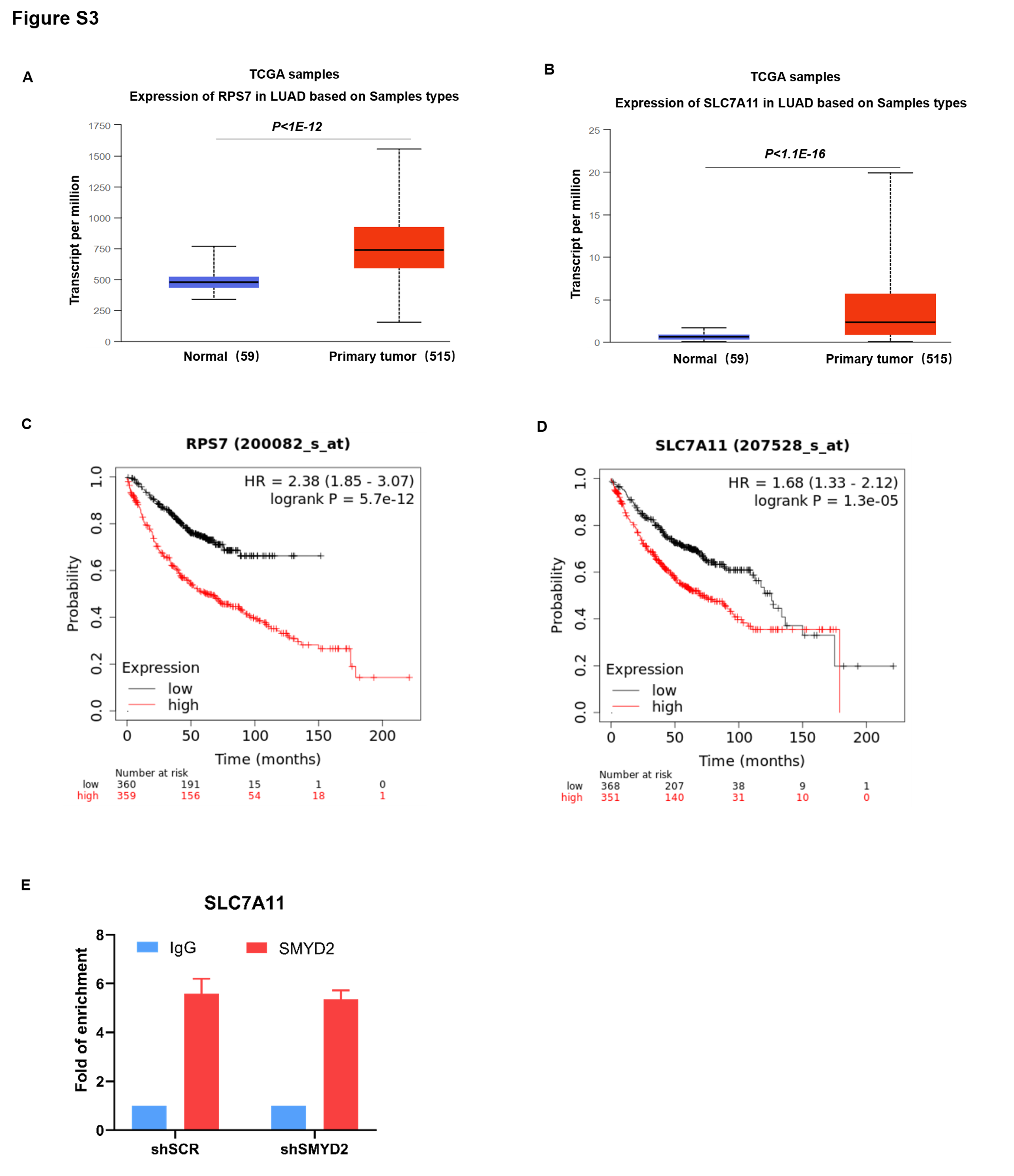

Supplement: Supplementary file 3 — Figure S3 [file 41419_2021_3720_MOESM3_ESM.png]
