# Supplementary material for: SMYD2 promotes tumorigenesis and metastasis of lung adenocarcinoma through RPS7
Source: Cell Death Dis. 2021 May 2;12(5):439. doi: 10.1038/s41419-021-03720-w (PMC8089105; doi:10.1038/s41419-021-03720-w)
Supplement: Supplementary file 4 — Table S1 [file 41419_2021_3720_MOESM4_ESM.pdf]

Table S1. siRNA and shRNA sequence used in the present study

	Sequence (5'-3')
shSCR	TTCTCCGAACGTGTCACGT
shSMYD2	CGATATTTCCTGATGTTGCAT
si-NC	UUCUCCGAACGUGUCACGUTT
si-SMYD2-1	GGAAGAUGCAAGCAGGCAUTT
si-SMYD2-2	GCUGUGAAGGAGUUUGAAUTT
si-SMYD2-3	GGAAGUAGCUCACGGCAAATT
si-RPS7	GCUGAAUAUUACGGCAGCUAATT
