# Supplementary material for: SMYD2 promotes tumorigenesis and metastasis of lung adenocarcinoma through RPS7
Source: Cell Death Dis. 2021 May 2;12(5):439. doi: 10.1038/s41419-021-03720-w (PMC8089105; doi:10.1038/s41419-021-03720-w)
Supplement: Supplementary file 6 — Table S3 [file 41419_2021_3720_MOESM6_ESM.pdf]

Table S3 Primers sequence used in the RT-qPCR and CHIP-qPCR assay

Gene	Strand	Sequence (5'-3')
RPS7	PROMOTER-FORWARD	CTCACGGCAGCCTCGACCTTC
RPS7	PROMOTER-REVERSE	GAGGCAGGAGGATCACTTGA
SLC7A11	PROMOTER-FORWARD	GTGACAGAAGTATAAAGTGTAG
SLC7A11	PROMOTER-REVERSE	TGCGACTGTTTTGTGAGTAGA
RPS7	FORWARD	GAAATTGTGGGCAAGAGAATCC
	REVERSE	AAGTTTCAACCTTGTGTTCCAC
SLC7A11	FORWARD	TTACCAGCTTTTGTACGAGTCT
	REVERSE	GTGAGCTTGCAAAAGGTTAAGA
SMYD2	FORWARD	GTAACGTGTACATGTTGCACAT
	REVERSE	GTAGAGTCTCCCTAGCTTCAAC
β-actin	FORWARD	ACAGAGCCTCGCCTTTGCCG
	REVERSE	TTGCACATGCCGGAGCCGTT
