# Supplementary material for: SMYD2 promotes tumorigenesis and metastasis of lung adenocarcinoma through RPS7
Source: Cell Death Dis. 2021 May 2;12(5):439. doi: 10.1038/s41419-021-03720-w (PMC8089105; doi:10.1038/s41419-021-03720-w)
Supplement: Supplementary file 7 — Table S4 [file 41419_2021_3720_MOESM7_ESM.pdf]

Table S4. Correlation analysis between expression of SMYD2 and clinical pathological characteristics of LUAD patients from TCGA database

Characteristics	n	SMYD2		X^2	*p* value
		High (%)	Low (%)		
T stage				4.290	0.232
T1	167	93 (55.7)	74 (44.3)		
T2	260	120 (46.2)	140 (53.8)		
T3	45	25 (55.6)	20 (44.4)		
T4	19	10 (52.6)	9 (47.4)		
N stage				2.792	0.425
N0	318	156 (49.1)	162 (50.9)		
N1	92	50 (54.3)	42 (45.7)		
N2	70	36 (51.4)	34 (48.6)		
N3	2	2 (100.0)	0 (0.0)		
TNM stages				7.154	0.067
I	263	131(49.8)	132 (50.2)		
II	117	65 (55.6)	52 (44.4)		
III	80	42(52.5)	38 (47.5)		
IV	26	7(26.9)	19(73.1)		
Age (years)				0.653	0.419
≤60	157	83 (52.9)	74 (47.1)		
>60	337	165 (49.0)	172 (51.0)		
Gender				6.814	0.009
Males	228	100 (43.9)	128 (56.1)		
Females	266	148 (55.6)	118 (44.4)		
