# Supplementary material for: SMYD2 promotes tumorigenesis and metastasis of lung adenocarcinoma through RPS7
Source: Cell Death Dis. 2021 May 2;12(5):439. doi: 10.1038/s41419-021-03720-w (PMC8089105; doi:10.1038/s41419-021-03720-w)
Supplement: Supplementary file 8 — Table S5 [file 41419_2021_3720_MOESM8_ESM.pdf]

Table S5. Correlation analysis between expression of SMYD2 and clinical pathological characteristics of LUAD patients from GEO (GSE31210) database

Characteristics	n	SMYD2		X^2	p value
		High (%)	Low (%)		
TNM stages				2.794	0.095
I	168	80 (47.6)	88 (52.4)		
II	58	35 (60.3)	23 (39.7)		
Smoking status				0.797	0.372
Yes	123	65 (52.8)	58 (47.2)		
No	123	58 (47.2)	65 (52.8)		
Age (years)				0.069	0.793
≤60	89	45 (50.6)	44 (49.4)		
>60	62	30 (48.4)	32 (51.6)		
Gender				0.065	0.798
Males	116	57 (49.1)	59 (50.9)		
Females	130	66 (50.8)	64 (49.2)		
