# Supplementary material for: SMYD2 promotes tumorigenesis and metastasis of lung adenocarcinoma through RPS7
Source: Cell Death Dis. 2021 May 2;12(5):439. doi: 10.1038/s41419-021-03720-w (PMC8089105; doi:10.1038/s41419-021-03720-w)
Supplement: Supplementary file 9 — Table S6 [file 41419_2021_3720_MOESM9_ESM.pdf]

Table S6. Correlation analysis between expression of SMYD2 and clinical pathological characteristics of LUAD patients from GEO (GSE13213) database

Characteristics	n	SMYD2		X^2	*p* value
		High (%)	Low (%)		
T stage				1.182	0.554
T1	79	38 (48.1)	41 (51.9)		
T2	13	6 (46.2)	7 (53.8)		
T3	25	15 (60.0)	10 (40.0)		
TNM stages				0.955	0.812
I	54	28 (51.9)	26 (48.1)		
II	50	24 (48.0)	26 (52.0)		
III	7	3 (42.9)	4 (57.1)		
IV	6	4 (66.7)	2 (33.3)		
Smoking index				2.629	0.105
<400	70	31 (44.3)	39 (55.7)		
≥400	47	28 (59.6)	19 (40.4)		
Age (years)				0.438	0.508
≤60	52	28 (53.8)	24 (46.2)		
>60	65	31 (47.7)	34 (52.3)		
Gender				1.918	0.166
Males	60	34 (56.7)	26 (43.3)		
Females	57	25 (43.9)	32 (56.1)		
