# Supplementary material for: SMYD2 promotes tumorigenesis and metastasis of lung adenocarcinoma through RPS7
Source: Cell Death Dis. 2021 May 2;12(5):439. doi: 10.1038/s41419-021-03720-w (PMC8089105; doi:10.1038/s41419-021-03720-w)
Supplement: Supplementary file 10 — Supplementary Figure Legends [file 41419_2021_3720_MOESM10_ESM.docx]

**Supplementary Figure Legends**

**Figure S1. The IC50 value of cells treated with AZ505 were detected and calculated.**

A and B, The growth of SPC-A1 and GLC-82 cells treated with AZ505 were detected under the concentration of 0,1nM,10nM,100nM,1000nM,10uM,20uM,40uM, 50uM,60uM,70uM,80uM,100uM by CCK8 assay. The calculated IC50 value of SPC-A1 and GLC-82 cells were 47.2μM and 53.4μM, respectively**.**

**Figure S2**. **The pathway enrichment assay of SMYD2 and the relationship assay of SMYD2 and RPS7/SLC7A11.**

A, The pathway enrichment assay was conducted according to the KEGG pathway database, and the top 20 pathways in the assay are enlisted in the figure. B and C, There were positive correlation between the expression of SMYD2 and RPS7/ SLC7A11 mRNA expression by GEPIA online analysis (r= 0.12, *p* = 0.0064 and r= 0.17, *p* < 0.001, Spearman correlation analysis). D, Through the analysis of PROGgeneV2 database, patients with SMYD2high/RPS7high had worse survival outcome, compared with SMYD2low/RPS7low patients from LUAD cohort (GSE13213).

**Figure S3. Upregulated expression of RPS7/SLC7A11 and their poor prognosis value in patients with LUAD from the online database.**

A and B, Box plots demonstrated RPS7 and SLC7A11 mRNA up-regulation in LUAD relative to normal lung tissues (data downloaded from ualcan: http://ualcan.path.uab.edu/). C and D, There were worse prognosis in patient with overexpression RPS7 and SLC7A11 in the LUAD cohort (data downloaded from Kaplan-Meier plotter: https://kmplot.com/analysis/index.php?p=service&start=1). E, ChIP-qPCR assays indicated that loss of SMYD2 did not diminish the enrichment on the promoter of the SLC7A11 gene.

**Supplementary Table Legends**

Table S1. siRNA and shRNA sequence used in the present study

Table S2. The mRNA expression data was obtained through the cBioPortal

Table S3. Primers sequence used in the RT-qPCR and CHIP-qPCR assay

Table S4. Correlation analysis between expression of SMYD2 and clinical pathological characteristics of LUAD patients from TCGA database

Table S5. Correlation analysis between expression of SMYD2 and clinical pathological characteristics of LUAD patients from GEO (GSE31210) database

Table S6. Correlation analysis between expression of SMYD2 and clinical pathological characteristics of LUAD patients from GEO (GSE13213) database
